# The Neuropeptide α-Calcitonin Gene-Related Peptide as the Mediator of Beneficial Effects of Exercise in the Cardiovascular System

**DOI:** 10.3389/fphys.2022.825992

**Published:** 2022-03-31

**Authors:** Tom Skaria, Johannes Vogel

**Affiliations:** ^1^School of Biotechnology, National Institute of Technology Calicut, Kerala, India; ^2^Zürich Center for Integrative Human Physiology, Institute of Veterinary Physiology, University of Zurich, Zurich, Switzerland

**Keywords:** exercise, cardiovascular health, αCGRP, myokines, migraine

## Abstract

Regular physical activity exerts cardiovascular protective effects in healthy individuals and those with chronic cardiovascular diseases. Exercise is accompanied by an increased plasma concentration of α-calcitonin gene-related peptide (αCGRP), a 37-amino acid peptide with vasodilatory effects and causative roles in migraine. Moreover, mouse models revealed that loss of αCGRP disrupts physiological adaptation of the cardiovascular system to exercise in normotension and aggravates cardiovascular impairment in primary chronic hypertension, both can be reversed by αCGRP administration. This suggests that αCGRP agonists could be a therapeutic option to mediate the cardiovascular protective effects of exercise in clinical setting where exercise is not possible or contraindicated. Of note, FDA has recently approved αCGRP antagonists for migraine prophylaxis therapy, however, the cardiovascular safety of long-term anti-CGRP therapy in individuals with cardiovascular diseases has yet to be established. Current evidence from preclinical models suggests that chronic αCGRP antagonism may abolish the cardiovascular protective effects of exercise in both normotension and chronic hypertension.

## Introduction

Cardiovascular diseases (CVD) including hypertensive heart disease, heart failure (HF), peripheral arterial disease, and stroke represent the main cause of morbidity and mortality. During the past decades, the number of deaths due to CVD increased steadily and it is estimated that CVD may cause more than 230 million deaths across the globe in 2030. Moreover, more than 10% of world’s annual total healthcare expenditure is spent for CVD (Roth et al., 2020; Amini et al., 2021).

Cardiovascular diseases is attributed to factors such as hypertension, unhealthy diet, heavy alcohol drinking, overweight, diabetes mellitus and physical inactivity (Amini et al., 2021). Numerous studies reveal that physical activity (PA) or reversal of sedentary lifestyle reduces the risk of cardiovascular events including HF and stroke (Virani et al., 2021). PA or exercise not only reduces the risk of CVD in healthy subjects but is also a central part of cardiac rehabilitation program recommended for subjects with CVD to reduce secondary events, hospital admissions and mortality (Virani et al., 2021). In fact, the ability of regular PA to increase cardiorespiratory fitness (CRF), an indicator of metabolic health, and thus to decrease CVD is well demonstrated (Kodama et al., 2009). Additionally, regular PA lowers the incidence of typical CVD risk factors such as systemic hypertension, hyperlipidemia, and diabetes mellitus. It also exerts direct beneficial effects on the structure of blood vessels and myocardium, improves autonomic balance and attenuates tissue damaging chronic proinflammatory responses (Fiuza-Luces et al., 2018). Thus, there is no doubt that exercise is beneficial in reducing CVD risk in healthy subjects and improving cardiovascular function in cardiovascular-compromised patients.

## α-Calcitonin Gene-Related Peptide: A Druggable Molecular Mediator of Cardiovascular Protective Effects of Exercise

Myokines, the peptides secreted by exercising skeletal muscles or nerve fibers innervating the contracting muscles, play important roles in mediating some of the structural and functional cardiovascular benefits of exercise (Pinckard et al., 2019). Myokines with prominent effects on cardiovascular system include C1q/TNF-related protein-1, neuron-derived neurotrophic factor, follistatin-like 1, IL-6 (Pinckard et al., 2019) and α-Calcitonin Gene-Related Peptide (αCGRP). Whether C1q/TNF-related protein-1, neuron-derived neurotrophic factor, follistatin-like 1 and IL-6 are indispensable for cardiovascular protective effects of exercise, i.e., whether their deficiency adversely affect the cardiovascular system during exercise, and their druggability still need to be defined by preclinical studies. αCGRP is a 37-amino acid druggable peptide released by nerve fibers of exercising skeletal muscles, trigeminal and perivascular nerves, endothelium, adipocytes, activated B lymphocytes, macrophages and keratinocytes. It differs from its isoform βCGRP by only three amino acids in humans. Sensory neurons preferentially express αCGRP (3–6-times higher than βCGRP) while in enteric neurons, βCGRP is upto 7-times more abundant than αCGRP [extensively reviewed by Russell et al. (2014) and Mulderry et al. (1988)]. αCGRP interacts with the receptor formed of calcitonin receptor-like receptor (CLR), receptor activity modifying protein (RAMP) and receptor component protein. Association of RAMP1 with CLR forms the specific receptor for CGRP whereas that of RAMP2 with CLR constitute the receptor for adrenomedullin [extensively discussed by Russell et al. (2014) and Liang et al. (2018)]. αCGRP, recently shown to mediate physiological cardiovascular adaptations to exercise in normotension and cardiovascular protective effects of exercise in chronic hypertension in preclinical models, is the focus of this review.

### Plasma and Muscle Tissue Concentrations of α-Calcitonin Gene-Related Peptide Increase During Exercise

A previous study observed highly increased plasma αCGRP concentrations in response to exercise in human runners before a training break, following 3 weeks of physical inactivity, and 2 and 4 weeks after recommencement of training, which showed positive correlation with heart rate (Schifter et al., 1995). Others found progressive increase in plasma αCGRP concentration during exercise in normal humans, hypertensives and diabetes patients and highest αCGRP concentration was observed at maximum exercise (Lind et al., 1996). A recent study has shown increased circulating αCGRP concentration in response to maximal exercise in 2/3 of study subjects who were healthy adults (Aracil-Marco et al., 2021). In an another study on subjects suffering from headache and headache-free subjects, endurance exercise increased plasma αCGRP concentration, and the post-run variation of circulating αCGRP was inversely related to running time (Tarperi et al., 2020). Moreover, graded exercise was shown to increase plasma αCGRP concentration in normal healthy subjects and those with coronary heart disease (CHD) with an additional, interesting finding that lower workload results in highest αCGRP concentrations in subjects with CHD. Increased oxygen demand in CHD might cause an earlier αCGRP release from sensory neurons during exercise (Lechleitner et al., 1994). Another study found increase in circulating αCGRP concentrations in close linear correlation to lactate in response to exercise in healthy sea level natives at sea level and subsequently after 24 h and 5 days in high altitude hypoxia. αCGRP release was not associated with increase in catecholamines or sympathetic vasoconstrictors like noradrenaline, rather it was strongly correlated with increased lactate levels only (Hasbak et al., 2002).

The major fraction of circulating immunoreactive αCGRP comprises the intact neuropeptide, suggesting exercise-induced αCGRP production and excluding the possibility that the increase in concentrations of immunoreactive αCGRP detected during exercise is caused by molecule degradation products (Schifter, 1991; Schifter et al., 1995; Hasbak et al., 2002). Both endurance and resistance training are capable of increasing αCGRP content in skeletal muscle tissues (Parnow et al., 2012) and increased αCGRP content is detectable in motor neurons and skeletal muscle tissues even 2 days after exercise (Homonko and Theriault, 1997).

### Putative Release Mechanism and Source of α-Calcitonin Gene-Related Peptide During Exercise

**FIGURE 1:**
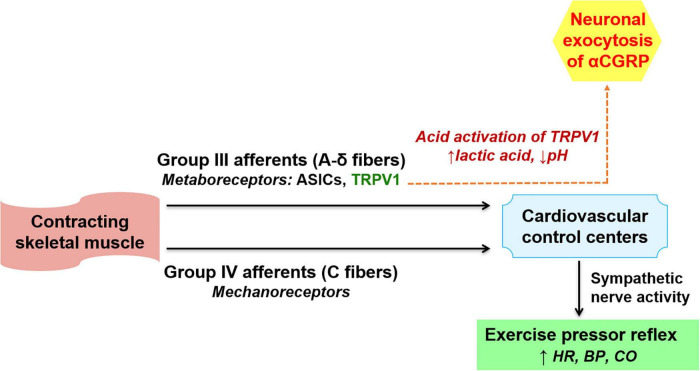
Mechanism of αCGRP release associated with exercise pressor reflex. During exercise, group III and IV afferent neurons within skeletal muscles are activated. This activates the sympathetic nervous system resulting in exercise pressor reflex (lower part of the figure). During exercise, tissue acidification (lactate production and drop in pH) induces αCGRP release from group III afferents through activation of transient receptor potential vanilloid subtype 1 (TRPV1) channels (dashed arrow, upper part of the figure). Post release, αCGRP entering the circulation may trigger physiological cardiac hypertrophy in normotension and mediate cardio-protection during pressure overload. ASICs, acid sensing ion channels, HR, heart rate, BP, blood pressure, CO, cardiac output.

During exercise, group-III and group-IV afferent neurons containing metaboreceptors and mechanoreceptors within the contracting skeletal muscles are activated. This leads to activation of sympathetic nervous system causing exercise pressor reflex that regulates blood pressure (BP) and heart rate changes during exercise (Cooper et al., 2016). The vanilloid receptor transient receptor potential vanilloid subtype-1 (TRPV1), a ligand-gated non-selective cation channel expressed abundantly in group III afferent neurons, is a metaboreceptor mediating exercise pressor reflex and is activated by capsaicin, noxious heat (43°C), and protons. Lactic acid formation and consequent drop in pH activates TRPV1, leading to both neuronal exocytosis of αCGRP and activation of the nociceptive transcription factor ‘cAMP response element-binding protein’ which in turn enhances αCGRP expression (Figure 1). On the other hand, αCGRP expression is inhibited by an inhibitor of Ca^2+^/calmodulin-dependent protein kinase (CaMK), thereby suggesting the involvement of CaMK in the downstream signaling associated with TRPV1-mediated αCGRP expression (Nakanishi et al., 2010). Lowering the pH or lactic acid alone triggered the release of αCGRP in rat spinal cord slices, and a combination of lactic acid with low pH, a condition mimicking strenuous exercise, caused more than additive stimulation of αCGRP release, suggesting lactic acid potentiates low pH-triggered αCGRP release (Wang and Fiscus, 1997). However, the underlying molecular mechanism involved in lactic acid-potentiated αCGRP release remain still undefined.

### Cardiovascular Protective Effects of Exercise-Activated α-Calcitonin Gene-Related Peptide Signaling in Physiological States

α-calcitonin gene-related peptide enhances the synthesis of acetyl choline receptors and activates Na^+^/K^+^ pump to counteract exercise-induced K^+^ depletion in myocytes. Therefore, an increase in circulating αCGRP concentrations may enhance both these processes at neuromuscular junctions during exercise (Schifter et al., 1995). Increase in αCGRP during exercise may also counteract the vasoconstrictor responses mediated by increased noradrenaline and neuropeptide-Y concentrations during sympathetic activation (Lind et al., 1996). αCGRP induces vasorelaxation through both endothelium- and NO-dependent and -independent pathways but does not regulate systemic BP in normal individuals (Russell et al., 2014). αCGRP may activate cAMP-dependent signaling pathways to mediate the cardiovascular protective effects of exercise. αCGRP protects cardiomyocytes from stress-induced apoptosis (Sueur et al., 2005) and triggers physiological cardiomyocyte growth *in vitro*, and produces positive inotropy and chronotropy (Bell et al., 1995; Al-Rubaiee et al., 2013; Schuler et al., 2014) which are the main cardiac adaptations to exercise. Exercise training caused fetal gene reactivation in the heart of adult, normotensive αCGRP knock out mice, resembling the pathological cardiac phenotype typically seen in hypertension. Moreover, treatment with the αCGRP receptor antagonist CGRP8-37 blunted exercise-induced physiological cardiac hypertrophy in normal mice. Furthermore, exercise performance was attenuated in normotensive, αCGRP knock out or CGRP8-37-treated wild-type mice but enhanced in transgenic mice overexpressing calcitonin receptor-like receptor (Table 1). This suggests that αCGRP augments maximum exercise capacity not only by acutely triggering positive chronotropy and inotropy but also by exerting direct hormonal protective effects on the heart to drive physiological cardiac hypertrophy (Schuler et al., 2014).

**TABLE 1:** Cardiovascular effects of exercise-activated endogenous, aCGRP signaling.

Subjects	Study setting/exercise intervention	Post exercise outcomes/findings on cardiovascular health
Humans	Normal, physically active healthy volunteers subjected to a graded exercise test up to exhaustion	Increased plasma CGRP concentration accompanied with enhanced cardiorespiratory fitness as indicated by increase in VO_2_, VCO_2_, carbohydrate oxidation rate and relative power in 2/3 of subjects (Aracil-Marco et al., 2021).

Mice	Adult mice globally deficient for aCGRP or CGRP receptor antagonist-treated WT mice with baseline hemodynamic variables including normal systemic blood pressure, subjected to treadmill exercise	Attenuated exercise performance, reactivation of myocardial foetal gene expression program (Schuler et al., 2014).
	
	Adult mice overexpressing the calcitonin receptor-like receptor with baseline hemodynamic variables including normal systemic blood pressure, subjected to treadmill exercise	Enhanced exercise performance (Schuler et al., 2014).
	
	Adult mice globally deficient for aCGRP or CGRP receptor antagonist-treated WT mice subjected to one-kidney one-clip model of chronic hypertension, subjected to 4 weeks of voluntary wheel running	Impaired survival, reduced voluntary wheel running activity, loss of beneficial effects of exercise on chronic hypertension-induced myocardial foetal gene reprogramming, pathological hypertrophic growth, fibrosis and function (Skaria et al., 2019).

Rats	6 weeks old rats underwent training protocol for 12 weeks and then treated with CGRP receptor antagonist prior subjected to single session endurance training	Attenuation of phospholipase C (PIPLC/IP3) pathway-mediated adipose tissue lipolysis during exercise (Aveseh et al., 2018).

In accordance with lipolytic effects observed in muscles after CGRP administration (Danaher et al., 2008), an acute increase in plasma αCGRP concentration enhanced adipose tissue lipolysis during exercise in rats, and this effect was inhibited by pretreatment with CGRP8-37 (Aveseh et al., 2018). This evidence supports the notion that exercise-induced αCGRP may exert hormonal effects (Table 1). An association of endogenous αCGRP with exercise-induced physiological cardiac hypertrophic growth is yet to be demonstrated in human subjects. However, a recent study has shown that healthy humans exhibiting an increase in circulating CGRP concentration also show a higher CRF, carbohydrate oxidation and work performance compared with those showing unaltered plasma CGRP concentrations post-exercise, suggesting CGRP release may be associated with physiological responses related to exercise (Aracil-Marco et al., 2021; Table 1).

### Cardiovascular Protective Effects of Exercise-Activated α-Calcitonin Gene-Related Peptide Signaling in Hypertension

A recent study has shown markedly reduced survival of αCGRP knock out mice following induction of chronic hypertension. Moreover, it was shown that inhibiting endogenous αCGRP signaling by gene knock out or treatment with CGRP8-37 does not further increase the systemic BP in mice with stable chronic hypertension. However, chronic hypertensive αCGRP knock out mice exhibited reduced voluntary running activity and cardiac function compared with chronic hypertensive wild type (wt) mice. Four weeks of voluntary exercise or systemic administration of αCGRP peptide increased cardiac function and suppressed myocardial fetal gene reactivation as well as cardiac fibrosis in chronic hypertensive wt mice. However, the cardioprotective effects of voluntary exercise in chronic hypertensive wt mice was abolished by CGRP8-37 treatment. Moreover, only systemic administration of αCGRP peptide but not voluntary running could suppress adverse myocardial remodeling and improve cardiac function in chronic hypertensive αCGRP knock out mice (Skaria et al., 2019; Table 1).

The aforesaid studies that investigated the role of endogenous αCGRP signaling in normotension (Schuler et al., 2014) and chronic hypertension (Skaria et al., 2019) used αCGRP knock out mice with unaltered, baseline systemic BP and calcitonin expression (Lu et al., 1999). Other αCGRP knock out mice with combined deletion of αCGRP and calcitonin, and altered baseline cardiovascular variables apparently due to calcitonin deficiency also exhibit impaired survival and cardiac function upon pressure stress (Supowit et al., 2005). Collectively, these findings from mice models suggest that even basal αCGRP concentration is important for survival and maintaining cardiac function in chronic hypertension, and myocardial protective effects of voluntary exercise in hypertension is mediated by endogenous αCGRP signaling. Moreover, these findings also suggest that αCGRP agonism may be a potential alternative or supplemental therapeutic strategy to mimic some of the therapeutically relevant cardioprotective effects of exercise in clinical conditions where patients are mobility impaired or exercise is otherwise contraindicated, i.e., αCGRP agonists could be used as an exercise mimetic.

Despite several independent previous studies reporting increased plasma αCGRP concentrations in response to exercise in humans (Schifter et al., 1995; Lind et al., 1996; Hasbak et al., 2003; Aracil-Marco et al., 2021), evidence for a direct effect of increased plasma αCGRP in mediating cardiovascular benefits of exercise and the molecular pathway(s) involved in these protective effects in humans is still scant and should be the focus of further studies. The intensity of exercise required to increase plasma αCGRP concentration, and whether there could be a correlation between circulating αCGRP concentrations with improvements in cardiovascular function in human subjects with chronic hypertension should be addressed.

A recent study employing endogenous αCGRP inhibition by gene knock out or by the αCGRP receptor antagonist BIBN4096 BS found that endogenous αCGRP can protect against elevated BP when nitric oxide synthase is inhibited. Moreover, it was shown that systemic αCGRP administration can reduce systemic BP and suppress pathological cardiovascular remodeling during states where vascular endothelial/nitric oxide system is dysfunctional. It suggests that αCGRP agonism may exert protective effects in pathological states associated with impaired nitric oxide production such as systemic hypertension (Argunhan et al., 2021). αCGRP exerts direct antifibrotic action by suppressing myofibroblast differentiation of cardiac fibroblasts through the activation of cAMP signaling pathway in the presence of hypertensive peptides such as Angiotensin-II (Skaria et al., 2019). αCGRP’s direct effects on inhibiting pathological collagen synthesis in cardiac fibroblasts (Skaria et al., 2019; Li et al., 2020) is in accordance with BP-independent protective effects of αCGRP in various cell types including cardiomyocytes (Bell et al., 1995; Sueur et al., 2005; Schuler et al., 2014).

Efficiency and druggability were the two major issues encountered while translating the previously discovered different, cardioprotective exercise signaling pathways to therapeutic regimen in the clinical setting (Vega et al., 2017). Intravenous administration of αCGRP improves myocardial contractility in patients with congestive HF (Gennari et al., 1990; Shekhar et al., 1991), thereby confirming αCGRP’s cardioprotective efficiency in clinical setting. The problem of the short plasma half-life (T_1/2_) of naive αCGRP peptide (<6 min) may be circumvented by using a new CGRP-analog (α-Analog) with extended T_1/2_ (>7 h) (Nilsson et al., 2016). The systemic administration of this acylated α-Analog with improved pharmacokinetics prevented end organ damage, and was well-tolerated in murine model of hypertension and HF (Aubdool et al., 2017).

### Potential Cardiovascular Adverse Effects of Anti-α-Calcitonin Gene-Related Peptide-Based Migraine Prophylaxis

It is well-established that αCGRP, through vasodilation and regulation of cerebrovascular nociception, plays a critical role in the pathophysiology of migraine (Russell et al., 2014), a highly devastating neurovascular disorder affecting upto 16% of the population worldwide. Therefore, there is un-waning interest in recently approved, αCGRP antagonism-based migraine prophylaxis. The monoclonal antibodies developed against αCGRP or its receptor (with long biological half-life of >45 days) are presumed to have no side effects due to toxic metabolites formation (Vollbracht and Rapoport, 2013; Bigal and Walter, 2014). Several phase-II and phase-III trials conducted to date could not find an increase in cardiovascular adverse effects following treatment with CGRP monoclonal antibodies compared with placebo (Sun et al., 2016; Skljarevski et al., 2018; Ashina et al., 2019; Ferrari et al., 2019; Silberstein et al., 2019; Xu et al., 2019). However, the safety of long-term CGRP antagonist therapy in cardiovascular-compromised patients (e.g., chronic primary hypertension) or those with major cardiovascular risk is not established yet.

A single iv. infusion of the anti-CGRP-receptor monoclonal antibody Erenumab did not affect exercise time in patients with stable angina due to coronary artery disease, prompting the authors to conclude that CGRP receptor blockade may not worsen myocardial ischemia (Depre et al., 2018). However, a serious concern about this study is that it does not provide insights to long-term effects of αCGRP antagonism. Such studies should observe patients for many years after administering antibodies to block αCGRP pathways. To the best of our knowledge, studies observing patients longer than 5 years (Ashina et al., 2019) haven’t been reported yet. Moreover, the study population of Depre et al. (2018) consisted of subjects with stable angina pectoris, often caused by stenosis of the epicardial conducting portions of the coronary artery. Actions of αCGRP are known to be limited in proximal, epicardial parts of the coronary artery bed whereas it is well-known that αCGRP is a potent vasodilator in the intramyocardial, smaller (distal) parts of the coronary artery bed. Another limitation of the study population is that 78% of the subjects were male despite the fact that majority of migraine sufferers are females. In female patients with angina pectoris, coronary artery disease is typically diffuse atherosclerosis with coronary microvascular dysfunction but without arteriographically-detectable stenosis. Therefore, it is likely that compared with males, females may respond differently to inhibition of αCGRP signaling. Furthermore, exercise treadmill test was conducted approximately 30 min after intravenous administration of the Erenumab, which may be a too short time period for the large molecular size Erenumab to reach the smooth muscle cells inside the vessels and bind to the CGRP receptor to effectively block it [extensively reviewed by Maassen van den Brink et al. (2018) and Rivera-Mancilla et al. (2020)].

α-calcitonin gene-related peptide deficient mice are resistant to develop diet-induced obesity and exhibit improved glucose handling and insulin sensitivity (Liu et al., 2017) whereas it was shown that treatment with CGRP receptor antagonist olcegepant does not affect systemic glucose and lipid metabolism but impair bone formation in mice model of diet-induced obesity (Köhli et al., 2021). This warrants further investigations on clarifying the role of endogenous CGRP signaling in regulating lipid and glucose metabolism in humans in physiological and pathological conditions and assessing bone status in patients treated with CGRP antagonists.

Strong evidence from preclinical models suggest that αCGRP, through nitric oxide- and vasodilation- dependent and independent mechanisms, protects against hypertension and hypertension-induced end organ damage including HF, and ischemic stroke (Aubdool et al., 2017; Skaria et al., 2019; Mulder et al., 2020). A recent single case study reported the development of a right thalamic infarction following the first dose of Erenumab in a 41-year-old woman with migraine without aura and with a history of long-term use of oral contraceptives (Aradi et al., 2019). As suggested by independent groups across the world, long term inhibition of αCGRP (e.g., for several years in humans) may severely impair cardiovascular function [Maassen van den Brink et al., 2016; Aubdool et al., 2017; Danser and Maassen van den Brink, 2017; extensively reviewed by Rivera-Mancilla et al. (2020)] and may invalidate therapeutically important beneficial cardiac effects of exercise in hypertensive subjects (Skaria et al., 2019).

## Conclusion

Exercise or regular PA undoubtedly reduces the risk of CVD and improves cardiovascular function in individuals with clinically diagnosed CVD. However, prevalence of PA did not increase considerably over the last decade, and physical inactivity was responsible for more than 800000 deaths in the year 2019. Moreover, subjects with contraindications for exercise or those with disabilities may fail to meet the PA requirements and thus may not derive the cardiovascular benefits of exercise which necessitates the need for an exercise mimetic. αCGRP could potentially be such an exercise mimetic because findings from preclinical models suggest that activation of endogenous αCGRP signaling may be one of the mechanisms by which exercise improves cardiovascular function in physiological conditions and cardiovascular diseases such as chronic hypertension. On the other hand, αCGRP’s cardiovascular protective effects raise concerns about the potential cardiovascular risks associated with chronic αCGRP antagonism, which has been recently approved by FDA for migraine prophylaxis.

## Author Contributions

TS and JV designed and wrote the manuscript. Both authors read and approved the final manuscript.

## Conflict of Interest

The authors declare that the research was conducted in the absence of any commercial or financial relationships that could be construed as a potential conflict of interest.

## Publisher’s Note

All claims expressed in this article are solely those of the authors and do not necessarily represent those of their affiliated organizations, or those of the publisher, the editors and the reviewers. Any product that may be evaluated in this article, or claim that may be made by its manufacturer, is not guaranteed or endorsed by the publisher.
